# A telomere-to-telomere assembly of *Oscheius tipulae* and the evolution of rhabditid nematode chromosomes

**DOI:** 10.1093/g3journal/jkaa020

**Published:** 2020-12-08

**Authors:** Pablo Manuel Gonzalez de la Rosa, Marian Thomson, Urmi Trivedi, Alan Tracey, Sophie Tandonnet, Mark Blaxter

**Affiliations:** 1 Tree of Life, Wellcome Sanger Institute, Cambridge CB10 1SA, UK; 2 Edinburgh Genomics, School of Biology, University of Edinburgh, Edinburgh EH9 3JT, UK; 3 Departamento de Genética e Biologia Evolutiva, Instituto de Biociências, Universidade de São Paulo (USP), São Paulo, SP 05508-090, Brazil

**Keywords:** Nigon elements, telomere to telomere, nematode, evolution, *Oscheius tipulae*

## Abstract

Eukaryotic chromosomes have phylogenetic persistence. In many taxa, each chromosome has a single functional centromere with essential roles in spindle attachment and segregation. Fusion and fission can generate chromosomes with no or multiple centromeres, leading to genome instability. Groups with holocentric chromosomes (where centromeric function is distributed along each chromosome) might be expected to show karyotypic instability. This is generally not the case, and in *Caenorhabditis elegans*, it has been proposed that the role of maintenance of a stable karyotype has been transferred to the meiotic pairing centers, which are found at one end of each chromosome. Here, we explore the phylogenetic stability of nematode chromosomes using a new telomere-to-telomere assembly of the rhabditine nematode *Oscheius tipulae* generated from nanopore long reads. The 60-Mb *O. tipulae* genome is resolved into six chromosomal molecules. We find the evidence of specific chromatin diminution at all telomeres. Comparing this chromosomal *O. tipulae* assembly with chromosomal assemblies of diverse rhabditid nematodes, we identify seven ancestral chromosomal elements (Nigon elements) and present a model for the evolution of nematode chromosomes through rearrangement and fusion of these elements. We identify frequent fusion events involving NigonX, the element associated with the rhabditid X chromosome, and thus sex chromosome-associated gene sets differ markedly between species. Despite the karyotypic stability, gene order within chromosomes defined by Nigon elements is not conserved. Our model for nematode chromosome evolution provides a platform for investigation of the tensions between local genome rearrangement and karyotypic evolution in generating extant genome architectures.

## Teaser

Here, González de la Rosa *et al*. explore the phylogenetic stability of nematode chromosomes using a new telomere-to-telomere assembly of the rhabditine nematode *Oscheius tipulae* generated from nanopore long reads. The authors resolve the 60 Mb genome into six chromosomal molecules and find evidence of specific chromatin diminution at all telomeres. They present a model for the evolution of nematode chromosomes through rearrangement and fusion of seven ancestral Nigon elements.

## Introduction

Linear chromosomes are basic elements of the organization of eukaryotic nuclear genomes. The number of chromosomes and the position of orthologous loci on them are generally conserved between closely related species, and conserved karyotypic elements have been identified even between distantly related taxa (Jaillon *et al.* 2004; Putnam *et al*. 2007; Nakatani *et al*. 2007). The evolutionary trajectories of genes, in terms of rate of drift and efficiency of selection, are influenced by their chromosomal location. For example, genes on sex chromosomes will be exposed as haploid in the heterogametic sex (whether X0, XY, or WZ), and their effective population size will be only 0.75 that of autosomal loci. More subtly, genes resident on longer chromosomes may be more affected by linked selection, as the number of recombination events is frequently limited to one per chromosome (Hammarlund *et al.* 2005) or chromosome arm, and the number of bases per centiMorgan will be larger in longer chromosomes. Gene evolution is also shaped by placement within chromosomes, with some regions, such as centromeres and subtelomeric regions experiencing higher rates of per-base and structural change (Rockman and Kruglyak 2009). On longer timescales, genes that have traveled together on single chromosomes might evolve to share dependence on long-range regulatory landscapes, such as the three-dimensional topologically associated domains that characterize chromosomal organization within interphase nuclei. For some sets of loci, such as HOX and paraHOX loci in most Metazoa, this constraint is evident between organisms that last shared common ancestors hundreds of millions of years ago (Krumlauf 2018).

Chromosome structural change is an important component of genome and species evolution (Sturtevant and Dobzhansky 1936). Chromosomal elements, sets of loci that have been colocated on the same linkage group for long periods of evolutionary time, have been identified in many taxa, including mammals (Band *et al.* 2000), Diptera (Bhutkar *et al.* 2008), Lepidoptera (d'Alençon *et al.* 2010), and Nematoda (Tandonnet *et al*. 2019). While many groups have deeply conserved karyotypes, species that have very different numbers of chromosomes or synteny relationships to closely related taxa will allow exploration of the constraints that act to retain chromosome number and gene content and also of the mechanisms that are involved in karyotypic evolution.

Most animals (Metazoa) have chromosomes with a defined centromere. However, several groups have holocentric chromosomes, where centromeric function is distributed across each chromosome (Albertson and Thomson 1982; Melters et al. 2012). *A priori*, holocentric organization might be thought to predispose a genome to increased rates of rearrangement both within and between chromosomes, as any chromosome fragment remaining after fission could still carry centromeric function, and fusions of chromosomes would not result in competing centromeres on the same molecule. However, Lepidoptera have holocentric chromosomes and generally conserved karyotypes (d'Alençon *et al.* 2010). The ancestral lepidopteran chromosome number is estimated to be 31, and while the genomes of some species that have fewer chromosomes, such as the genus *Heliconius* (where *n* = 21), can be modeled through a series of simple fusions (d'Alençon *et al.* 2010), others, such as *Pieris napi* (the green-veined white butterfly; *n* = 25), exhibit extensive rearrangement, including presumed ancestral linkage group fragmentation and fusion (Hill *et al.* 2019). Thus, to distinguish the conservation of chromosome number *per se* from conservation of linkage groups, and to define the patterns and processes involved in changes in karyotype, complete, telomere-to-telomere chromosomal assemblies are needed (Hill *et al.* 2019).

Nematode chromosomes are also holocentric. The model nematode *Caenorhabditis elegans* (Rhabditomorpha, Rhabditina, Rhabditida; see De Ley and Blaxter 2002) has *n* = 6 and an X0 sex determination mechanism. In *C. elegans*, each chromosome has a single, telomeric or subtelomeric meiotic pairing center (McKim *et al*. 1988, 1993; Zetka and Rose 1992). Correct pairing at these sites is essential for synapsis and crossing over (MacQueen *et al.* 2005; Tsai and McKee 2011). Given that crossover is limited to properly synapsed homologous chromosomes (Lui and Colaiácovo 2013; Cahoon *et al.* 2019), pairing centers could play a similar role to centromeres in defining the number of chromosomal units and suppression of karyotype evolution (MacQueen *et al.* 2005; Rog and Dernburg 2013). In the order Rhabditida, *n* = 6 is the commonest karyotype (Supplementary Table S1) (Walton 1959), but *n* varies between 1 (*e.g. Diploscapter coronatus*, closely related to *Caenorhabditis*) (Fradin *et al.* 2017) and >50 (*e.g. Meloidogyne* polyploids) (Triantaphyllou 1963).

In Lepidoptera, the conserved karyotype is associated with the conservation of gene placement and gene order on each chromosome (*i.e.* there is conserved macro- and microsynteny) (Pringle *et al*. 2007). In contrast, in *Caenorhabditis* species (all with *n* = 6) while orthologous genes are overwhelmingly located on orthologous chromosomes, local gene order is very different between species (Stein *et al*. 2003; Stevens *et al*. 2020; Teterina *et al*. 2020). This pattern, of conservation of macro-synteny in the absence of microsynteny, is also observed in comparisons of *Caenorhabditis* to other genera (Doyle *et al.* 2019).

Some nematodes have different karyotypes in their somatic cells compared to their germline. This process involves scission of germline chromosomes and loss of germline material and is called chromatin diminution (Wang and Davis 2014). Chromatin diminution has been observed in several metazoan taxa, including chordates (Kinsella *et al.* 2019) and insects (Goday and Esteban 2001), and is involved in the generation of the ciliate macronucleus (Rzeszutek *et al*. 2020). The process of diminution is best understood in *Ascaris suum* (Ascarididomorpha, Spirurina, Rhabditida), where the germline has *n* = 24 chromosomes but somatic cells have *n* = 36 (Wang *et al*. 2020). In *A. suum*, the breakage events affect some but not all of the X chromosomes (*A. suum* has five X chromosomes) and autosomes, and breakage and neo-telomere addition happen in a defined area of the chromosome, but not at a precise base position. Related ascarididomorph nematodes also display diminution. Chromatin diminution has also been described in the tylenchomorph nematode *Strongyloides papillosus*, where loss of a specific internal fragment of one copy of the X chromosome generates a haploid region that is associated with males (*i.e.* sex determination in *S. papillosus* is effectively XX:X0, but the nullo-X is determined through specific deletion) (Albertson *et al.* 1979).

Previously, we proposed the existence of seven ancestral chromosome elements in rhabditine nematodes, named Nigon elements, and used this model to understand chromosome evolution in a few genome-sequenced Rhabditina (Tandonnet *et al.* 2019). The lack of chromosomally complete genomes limited the power of the model. Here, we present an improved chromosomal genome assembly of *Oscheius tipulae* (Rhabditomorpha, Rhabditina, Rhabditida). *O. tipulae* is a satellite genetic model organism that is used to understand the evolution of developmental systems such as the specification of the nematode vulva, and the genome sequence is required to underpin detailed genetic mapping (Besnard *et al.* 2017). The new telomere-to-telomere assembly allowed us to identify unexpected features of chromatin diminution at the telomeres of each chromosome. We used the *O. tipulae* genome and other chromosomally complete nematode genomes to fully define sets of orthologous genes associated with ancestral Nigon elements. This analysis allowed us to map ancient chromosomal fusions and scissions and identify a set of genes that is always associated with the X chromosome in both X0 and XY taxa in the order Rhabditida.

## Materials and methods

### Nematode culture, DNA extraction, and QC

*O. tipulae* strain CEW1 (Evans *et al.* 1997) was obtained from Marie-Anne Félix (Institute of Biology of the Ecole Normale Supérieure, Paris) and cultivated at 20°C in 5-cm nematode growth medium lite plates seeded with *Escherichia coli* HB101 (Stiernagle 2006). Nematodes were washed from culture plates using an M9 buffer supplemented with 0.01% Tween 20. Nematodes were pelleted by low-speed centrifugation, and 100 µl of samples transferred with minimal supernatant to 1.5-ml LoBind Eppendorf tubes. Nematodes were lysed by the addition of 600 µl of Cell Lysis Solution (Qiagen) and 20 µl of proteinase K (20 µg/µl) and incubated at 56°C with mixing at 300 rpm for 4 h. RNA was digested by adding 5 µl of RNAse Cocktail Enzyme Mix (Invitrogen) and incubating at 37°C for 1 h. Protein was precipitated by adding 200 µl of ice-cold Protein Precipitation Solution (Qiagen), gentle mixing and incubation on ice for 10 min. The precipitate was pelleted by centrifugation for 30 min at 4°C at 15,000 rpm. The supernatant was transferred to a LoBind tube and nucleic acids were precipitated by the addition of 600 µl of ice cold isopropanol, mixing by inversion, and incubation on ice for 10 min. Nucleic acids were pelleted by centrifugation at 4°C at 15,000 rpm. The supernatant was discarded, and the pellet washed twice using 600 µl of 70% ethanol. The pellet was air-dried for 5 min and resuspended in 20 µl of elution buffer. DNA recovery and quality was assessed by Qubit fluorimetry, Tapestation genomic Screentape (Agilent), and pulsed field gel electrophoresis using a Pippin Pulse instrument. The sample used for sequencing had a DNA concentration of 120 ng/µl, a DNA integrity number of 9, and an RNA concentration of 9 ng/µl.

### Genomic sequencing on Oxford Nanopore PromethION

To generate fragments of a suitable size range for Oxford Nanopore PromethION sequencing, high molecular weight DNA was diluted to a concentration of 25 ng/µl and fragmented to an average peak size of 25 kb using a Megaruptor-2 instrument (Diagenode). Small fragments <1 kb were removed and the DNA concentrated using bead purification (0.4× volumes of Ampure-XP beads). Two aliquots of 1 µg of sheared *O. tipulae* DNA and control DNA (lambda 3.2-kb fragment) were subjected to DNA damage repair (NEBNext FFPE DNA Repair Mix; New England Biolabs) followed by DNA End Repair (NEBNext Ultra II End Repair/dA-tailing Module; New England BioLabs). A second 0.4× volume Ampure-XP bead clean-up was carried out, and the DNA was eluted in sterile distilled H_2_O. Oxford Nanopore sequencing adapters were ligated to 750 ng of the recovered, end repaired DNA using the Ligation Sequencing kit (SQK-LSK-109; Oxford Nanopore) and NEBNext Ligation Module (New England BioLabs). Following a further 0.4× volume Ampure-XP purification, the recovered DNA (16.25 fmol) was loaded onto a R9.4.1 PromethION flow cell following the manufacturer’s instructions and a 60-h sequencing run was initiated.

Raw reads were basecalled using Guppy (see Supplementary Table S2 for software tools and settings used). The resulting dataset of 8.8 M reads spanned 108.4 Gb and had a read N50 of 19.1 kb (Supplementary Figure S1A and Supplementary Table S3). To identify sequence contamination, we assembled a custom kraken2 database composed of bacteria, fungi, human, UniVec core, and a selection of nematode genomes including the previous *O. tipulae* assembly (Supplementary Table S4). The vast majority of reads (99.5%) were classified. One-fifth (19.1% of the total bases) were classified as Nematoda, and the remainder as Proteobacteria (97.5% of these belonging to *Escherichia*; these likely derive from bacterial food). Minor human, fungal, and other bacterial contamination was also present (Supplementary Table S5). We removed reads classified as Bacteria, Chordata, Ascomycota, Basidiomycota, or Microsporidia. The remaining data spanned 20.7 Gb in 2.8 million reads with a read N50 of 14.4 kb (an estimated ∼340-fold coverage).

### Genome assembly and polishing

Several different assembly strategies were explored (Supplementary Table S6). Flye (Kolmogorov *et al.* 2019) in metagenome mode with the whole long read set yielded a chromosome level assembly of *O. tipulae* together with contaminant species (Supplementary Figure S1B). This assembly was polished using Racon (Vaser *et al*. 2017) and medaka using the decontaminated read set. For Pilon polishing (Walker *et al*. 2014), previously published Illumina reads (Besnard *et al.* 2017) were trimmed with BBDuk (Bushnell 2017) and aligned with BWA-MEM (Li and Durbin 2009). We derived the chromosome assembly nOti 3.1 by stitching back the two sequences of chromosome I with RaGOO (Alonge *et al.* 2019) using the unpolished assembly as a reference. An alternate assembly was obtained using Flye in metagenome mode with only 40× Canu-corrected, decontaminated reads, followed by Racon and Pilon polishing. This assembly had higher BUSCO completeness than nOti 3.1 but was more fragmented. We derived a new consensus, nOti 3.2, from nOti 3.1 and the decontaminated-read Flye assembly using gap5 (Bonfield and Whitwham 2010) giving the decontaminated read assembly a 100× relative weight. The resulting contigs were assigned chromosome names by the longest match to contigs in nOti 2.0, which were previously assigned to chromosomes (Besnard *et al.* 2017). Alignment of the raw reads against nOti 3.2 showed that the nuclear genome had an average per-base coverage of 334-fold (standard deviation of 198). The initial Oti_chrV sequence had the highest coverage and coverage heterogeneity (354-fold, SD 478) due to collapse of the ribosomal RNA cistron between positions 7,413,040 and 7,440,271 (see below; Supplementary Table S7).

We curated the nOti 3.2 assembly by examining read coverage across the genome. A gap5 (Bonfield and Whitwham 2010) database was built from a 200× sub-sample of the longest PromethION canu-corrected reads. We noticed that all the chromosomes were characterized by a shorter majority sequence (80% of the average coverage depth) and a longer minority sequence (20% coverage depth). Both of these sequences terminated in telomeric repeat (long tandem repeats of TTAGGC). These alternate telomeric repeat addition sites appeared not to be artifacts because long reads supporting both versions were anchored in unique sequence. Previous Illumina short read data also identified similar major and minor components of the chromosome ends and supported the same telomeric repeat addition sites. We manually extended all reads containing soft-clipped telomeric repeat sequence and then used the gap5 realign function to produce a new consensus from them. The left hand end of the Oti_chrIV sequence produced directly by the assembler was characterized by an artificial sequence as evidenced by a lack of reads that mapped to it and all reads being soft-clipped either side of it. This sequence was replaced with realigned, soft-clipped sequence from the adjacent mapped reads. Restoration of these soft-clipped data identified telomeric repeat at both ends of each chromosome. We estimated the size of the highly collapsed ribosomal RNA cistron repeat on Oti_chrV. The rRNA cistron repeat was estimated to be 6.8-kb long and to be present in 117 copies, based on its coverage by Illumina short reads. The left hand side of the rRNA cistron repeat terminated in an obviously mispredicted sequence, which was dealt with as for the Oti_chrIV telomere sequence. We extended the reads at the junctions into and out of the rRNA cistron repeat sequence to a minimum depth of 2 reads. We joined these flanks together using 760128 “N” characters to match 111 additional copies of the 6.8-kb rRNA cistron repeat. We polished this assembly via three rounds using freebayes through snippy (Seemann 2014) with Illumina reads. We identified spliced leader RNA (SL) and 5S rRNA loci using Rfam models (Nawrocki *et al*. 2015) (Supplementary Figure S2).

### Genome annotation

We created a repeat library for our assembly following published protocols (Coghlan *et al.* 2018). Briefly, we identified repetitive sequences with RepeatModeler2 (Flynn *et al.* 2020), transposons with TransposonPSI (Haas 2007), and Long Terminal Repeats (LTR) with LTRharvest (Ellinghaus *et al.* 2008). We discarded TransposonPSI predictions shorter than 50 bp. We filtered out LTRharvest predictions that lacked PFAM (Finn *et al.* 2016) and GyDB (Llorens *et al.* 2011) hidden Markov model domain hits using LTRdigest (Steinbiss *et al*. 2009). The three prediction sets were classified with RepeatClassifier and merged into a single library. Sequences were clustered if they had more than 80% identity using USEARCH (Edgar 2010). This repeat library, together with the CONS-Dfam_3.1-rb20181026 database (Hubley *et al.* 2016), was used by RepeatMasker to annotate the repetitive regions in the assembly. We predicted protein-coding genes using GeneMark-ES (Lomsadze *et al.* 2005). We explored helitron predictions using HelitronScanner (Xiong *et al*. 2014). For nucleotide and protein-coding gene comparisons in the telomere extensions, we used BLAST+ (Camacho *et al.* 2009), ClustalW (Thompson *et al*. 2002), and JalView (Waterhouse *et al*. 2009). We identified genes and other sequence features of telomeric extensions using BLAST similarity searches and hidden Markov model searches using Dfam helitron models (Wheeler and Eddy 2013). Images were generated using the circos toolkit (Krzywinski *et al.* 2009).

### Comparison to genomes of other Rhabditida and identification of loci supporting Nigon elements

We assessed chromosomal assemblies and annotations of seventeen rhabditid nematode species (Supplementary Tables S8 and S9). For 15 species, we extracted the longest protein per gene from genome annotation GFF3 files using AGAT (Dainat 2020). Orthogroups were identified by Orthofinder (Emms and Kelly 2015, 2019) using these proteomes. Orthogroups were filtered using Kinfin (Laetsch and Blaxter 2017) to identify fuzzy single-copy orthologs in at least 12 of the 15 species compared (see Supplementary File S1).

We identified loci that are found on the same chromosomal unit in multiple species using a subset of nine genome assemblies that are resolved into chromosomes: *Auanema rhodensis* (Tandonnet *et al.* 2019), *Brugia malayi*(Foster *et al.* 2020), *Haemonchus contortus*(Laing *et al.* 2016), *Meloidogyne hapla*(Opperman *et al*. 2008), *Onchocerca volvulus*(Cotton *et al.* 2016), *O. tipulae*, *Pristionchus pacificus*(Rödelsperger *et al*. 2017), *Steinernema carpocapsae*(Serra *et al*. 2019), and *Strongyloides ratti*(Nemetschke *et al*. 2010b). For *M. hapla*, contigs were grouped into chromosomes according to the genetic linkage map (Opperman *et al*. 2008). For *S. carpocapsae*, only the X chromosome was assembled to completeness, while the four autosomes were present as 12 unassigned scaffolds. To reduce phylogenetic bias, we used a single *Caenorhabditis* species (*C. elegans*).

The chromosomal location of each single-copy ortholog in each species was extracted from genome GFF3 files and collated in an ortholog-chromosomal allocation matrix by species. Scaffolds “a” and “b” of *O. volvulus* chromosome 1 were treated as a single chromosome, as were the *S. ratti* X chromosome scaffolds. We calculated the Dice distance (represented as 1-Dice distance) between all ortholog pairs based on the pattern of their chromosomal allocation between species. A pair of genes found on the same chromosome in all the species would have a similarity of 1, while a pair found on different chromosomes in all the species would have a similarity of 0. This matrix was clustered with CLARA (Clustering Large Applications) using an expected number of clusters, *k*, from 1 to 10 (Supplementary Figure S3). CLARA chooses independent subsamples of the data, applies PAM (partitioning around medoids) to each of these, and selects the medoids with the minimal costs. In this case, CLARA identified the medoids (center of the clusters) by applying PAM to five independent subsamples each containing 10% of the orthologous loci. To identify the best number of clusters, we assessed average silhouette values that will be higher when the average loci are closer to members of the same clusters than to loci of other clusters. The highest silhouette value was found at *k* = 7 when using PAM over the whole matrix or CLARA clustering (Supplementary Figure S3, A and D). CLARA was used because it reduced computation time. Similarly, seven clusters were identified by the t-Distributed Stochastic Embedding (t-SNE) plot. These clusters were also found when using different perplexity values, which can be interpreted as the number of effective nearest neighbors, from 30 to 1000 (Supplementary Figure S4). Orthologs were assigned to clusters as long as at least seven taxa agreed on their colocation with other orthologs in the cluster. Cluster numbers were assigned to putative element groups when they contained more than 20 single-copy orthologs and allocated to Nigon element labels according to our previous classification (Tandonnet *et al.* 2019).

To visualize the genomes of *A. suum*(Wang *et al.* 2020), *Bursaphelenchus xylophilus*(Dayi *et al.* 2020), *B. okinawaensis* (Sun *et al*. 2020), and *Heterorhabditis bacteriophora* (Bai *et al.* 2013), their orthologs to *C. elegans* genes were identified by best reciprocal best hit. Given the high duplication of single copy oirthologs in the assemblies of *Diploscapter pachys* (Fradin *et al.* 2017) and *D. coronatus* (Hiraki *et al.* 2017), we selected one of the multiple proteins with hits to *C. elegans* genes having a blastp *e*-value lower than 1e−6 and giving preference to proteins found in longer scaffolds. These genes were assigned to Nigon units according to the assignment of the *C. elegans* ortholog.

### KEGG pathway enrichment analysis

We assessed functional enrichment of KEGG pathways among each Nigon defining loci set using the *C. elegans* representatives through gProfiler (Raudvere *et al*. 2019). We downloaded the *C. elegans* gene KEGG annotations using the R package KEGGREST (Tenenbaum 2019). We used a Fisher exact test controlling the false discovery rate by Benjamini–Hochberg *P*-value correction to assess pathway enrichment using the 2175 *C. elegans* Nigon defining loci as comparator.

### Data availability

The raw PromethION data are available in INSDC under accession SRR12179520 associated with the BioSample SAMN15480678. The genome assembly has been deposited in INSDC with the accession number GCA_013425905.1. The data used to generate the Tables and Figures are available at https://docs.google.com/spreadsheets/d/1gO4j4jSgSYQ_Aofl59RdGbwHrz2L2loWfrDIyyRaohw/edit?usp=sharing. Scripts and intermediate files associated with this study are available at https://github.com/tolkit/otipu_chrom_assem and doi:10.5281/zenodo.4265461 under a GPL-3.0 License. Protein sequences representative of each Nigon loci are found in Supplementary File S3.

Supplementary material is available at figshare DOI: https://doi.org/10.25387/g3.12982394.

## Results

### A chromosomal assembly of *O. tipulae* CEW1 from Oxford Nanopore PromethION data

Our previous assembly of *O. tipulae* CEW1 had a contig N50 of less than 1 Mb and was resolved to chromosomes using genetic map data, which necessarily excluded contigs with no mapped loci and could not orientate contigs mapped through a single genetic locus (Besnard *et al.* 2017). To generate a telomere-to-telomere, chromosomally complete *O. tipulae* genome, we resequenced CEW1 using the Oxford Nanopore Technologies PromethION platform, which generates large numbers of long reads from single molecule. After removing reads from bacterial contamination, we retained approximately 340-fold coverage of the expected 60 Mb genome (Supplementary Table S5). We explored assembly options using a range of tools and polished the assemblies to correct remaining errors with previously obtained Illumina short read data. Twelve assemblies were generated, with nematode sequence contiguities ranging from 7 to over 900 contigs (Supplementary Table S6). The most contiguous assemblies had seven contigs, six with multi-megabase lengths and one corresponding to the mitochondrial genome. Each of the assemblies contained about 90% of the BUSCO set of conserved nematode orthologs. An assembly generated using Flye (Kolmogorov *et al.* 2019) in metagenome mode, combining data from decontaminated and full read sets, scored best using BUSCO and contiguity measures. We curated the genome by circularizing the mitochondrion and identifying and correcting two remaining issues in the nuclear sequence. The ribosomal RNA repeat cistron was present as a collapsed and jumbled sequence, as was the case until recently with the *C. elegans* complete genome sequence. A gap representing the estimated span of the repeat was inserted. While the 5S rRNA and spliced leader 1 RNA loci are present as a tandem repeat in *C. elegans* and related nematodes, the 5S and SL1 RNA genes were not clustered in *O. tipulae* (Supplementary Figure S2).

**Figure 1:**
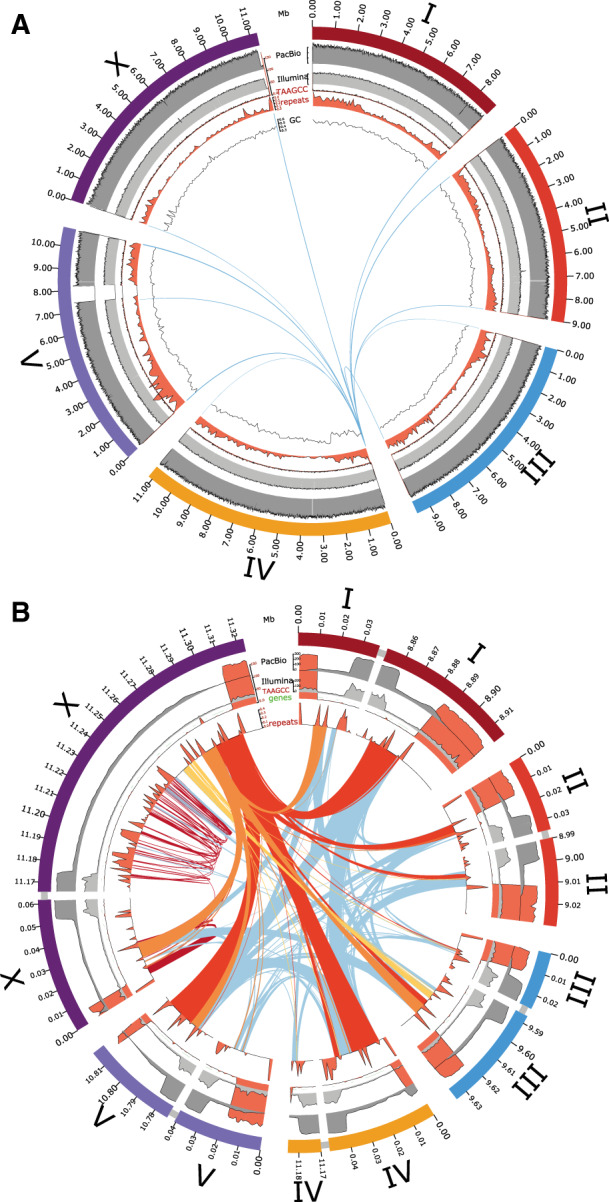
Subtelomeric extensions on the chromosomes of *O. tipulae* CEW1. (A) Circos plot of the *O. tipulae* CEW1 genome showing (from outside to inside) the chromosomes with a scale in Mb, coverage in PromethION reads (dark grey), coverage in Illumina short reads (light grey), count of telomeric repeats per 10 kb (red), density of repeats (red) and GC proportion in 10-kb windows (line plot). The inner arcs link all significant nucleotide sequence (blastn) matches between the presumed near-complete copy of the helitron on the left end of Otip_IV (with an open reading frame of 3895 amino acids, spanning 26-kb of the telomere extension) and the rest of the genome. (B) Circos plot of the low-coverage subtelomeric extension sequences of *O. tipulae* CEW1, with 10 kb of flanking chromosome, showing (from outside to inside) the chromosomes with a scale in 100 kb, coverage in PromethION reads (dark grey), coverage in illumina short reads (light grey), count of telomeric repeats per 10 kb (red), density of repeats (red), and predicted coding genes (green). Arcs are drawn linking significant blastn matches between three sequential helitron-like components from the right telomere extension of chromosome X (yellow, orange and red links), between repeats limited to within Otip_X R (dark red), and between all other telomeric extension sequences (light blue).

The second issue concerned the chromosomal termini. Only four of the 12 ends of contigs in the initial assembly ended in telomeric repeat sequence ([TTAGGC]_*n*_, the same repeat as found in *C. elegans*). We were able to extend each contig end into telomeric repeat using long reads that overlapped the unique sequence at each end of each contig. The assembly was thus judged complete, telomere to telomere. The six longest contigs corresponded to the six genetically defined linkage groups of *O. tipulae*, and we named these Otip_I, Otip_II, Otip_III, Otip_IV, Otip_V, and Otip_X, following the previously defined chromosome nomenclature (Besnard *et al.* 2017). However, at each telomere, we identified two independent and specific sites where there was transition from unique sequence to tandem hexamer telomere repeat: an internal site supported by 80% of the PromethION reads, and an external site, supported by a minority (20% of reads). The extension sequences were confirmed by relatively even, 60-fold coverage of PromethION reads and by matching reads in Illumina short-read data, which also showed a majority-short and minority-long distribution (Figure 1). These telomere repeat addition sites define three components on each chromosome: a central portion, present in all copies, and two subtelomeric extension sequences, from the regions at the left and right ends, present in a minority of copies. In turn, this implies that *O. tipulae* chromosomes are each found as long-form (carrying the extensions) and short-form (lacking the extensions) versions. We cannot exclude the possibility that absence of the extensions on the left and right ends of each chromosome is determined independently, but the very similar proportional coverage of all extensions strongly suggests that chromosomes are either all short form or all long form in each cell.

The subtelomeric extensions ranged from 4 to 133 kb (excluding the telomeric hexamer repeats) and totaled 349 kb (Table 1 and Figure 1B). The assembly included an additional 150 kb of telomeric repeat. The subtelomeric extension sequences had an unremarkable GC proportion (45–49%) and contained unique sequence, including predicted protein-coding loci that had support from uniquely mapping transcript evidence (Besnard *et al.* 2017). Notably, the telomere extensions contained repeat families that were largely limited to the extensions (Table 1 and Figure 1B). One predicted protein-coding gene overlapped the internal telomere addition site [gene 632t, encoding a neprilysin M13 metallopeptidase homolog, on Otip_V right end (Otip_V R)]. This gene prediction appears to represent a full-length locus, as it aligns well with *C. elegans* orthologs, and would therefore be predicted to be nonfunctional in the short-form chromosome. The repeat sequences unique to the subtelomeric extensions were predicted to encode protein-coding genes that had similarities to helitron transposon genes (Figure 1). The longest helitron-like predicted gene, 9690_t on Otip_IV left end (Otip_V L) (3985 amino acids) contains domain matches to Pif1-like, ATP-dependent DEAH-box DNA helicases. Sequences similar to this putative helitron-like gene are present on 11 of the 12 telomere extensions (it was absent from the Otip_I L extension; Figure 1A and Table 1). A single additional match was found on Otip_V, near the rRNA cistron repeat. Scanning of the genome for helitron-like sequences using RepeatFinder (Tarailo-Graovac and Chen 2009) and HelitronScanner (Xiong *et al.* 2014) identified many additional, distinct helitron-like sequences, scattered across all the chromosomes. However, the telomere-associated helitron-like elements scored poorly in these searches, suggesting that they are a new, perhaps distinct family.

**Table 1:** Telomeric extension sequences in *O. tipulae*

Chromosome	Arm	Extension span (bp)	**Subtelomeric extension start site** ^a^	**External TTAGGC telomere repeat addition site** ^a^	External TTAGGC telomere repeat span (bp)
Otip_I	L	16,822	27,184	10,363	10,362
	R	24,306	8,885,185	8,860,879	31,405
Otip_II	L	10,122	25,180	15,059	15,058
	R	10,413	9,010,174	8,999,761	19,801
Otip_III	L	4060	16,298	12,239	12,238
	R	25,188	9,621,518	9,596,330	13,932
Otip_IV	L	34,114	397,82	5669	5668
	R	4711	11,184,555	11,179,844	463
Otip_V	L	23,599	30,663	7065	7064
	R	29,106	10,812,563	10,783,457	4916
Otip_X	L	32,697	51,845	19,149	19,148
	R	133,498	11,309,184	11,175,686	14,155
Total		348,636			154,210

a Base position with reference to the full chromosome sequence length (including telomere hexamer repeat and telomere extensions). This position is the site of addition of telomeric hexamer repeat in the short-form chromosomes.

The final nuclear genome assembly was a significant improvement over the previous reference in contiguity and completeness (Besnard *et al.* 2017) (Table 2). The 253 contigs of the previous assembly were super-scaffolded using genetic map information. All but eight of these previous assignments were replicated in the new assembly, and the data underlying the new assembly affirmed the eight new assignments. The longest contigs from the previous assembly tended to be found in the centers of the chromosomal contigs, and the ends of the new chromosomal contigs were represented by multiple shorter contigs in the previous assembly (Supplementary Figure S5). The proportion of complete nematode BUSCO loci (nematoda_odb10) identified in both assemblies was 90%. Close analysis of the assemblies identified candidates for some of the 254 apparently missing orthologs. We note that similar low BUSCO completeness scores were recorded for the closely related nematode *A. rhodensis* (Supplementary Table S9).

**Table 2:** Metrics of the *O. tipulae* CEW1 genome assembly

Species	*Oscheius tipulae CEW1 nrOscTipu1.3*	*Oscheius tipulae CEW1 Ot_2.0*	*C. elegans PRJNA13758 WBPS12*
Reference	This work	Besnard *et al.* (2017)	From WormBase^a^
Genome			
Span (Mb)	60.9	59.5	100.3
Number of scaffolds	6 (+MT)	191	6 (+MT)
Scaffold N50 (Mb)	10.8 (chromosomal)	1.2	17.5 (chromosomal)
Number of contigs	6 (+MT)	256	6 (+MT)
Contig N50 (Mb)	10.8 (chromosomal)	0.7	17.5 (chromosomal)
Genome BUSCO^b^	C: 90.7% [S: 88.7%, D: 2.0%], F: 1.8%, M: 7.5%	C: 91.6% [S: 89.0%, D: 2.6%], F: 1.6%, M: 6.8%	C: 99.4% [S: 98.9%, D: 0.5%], F: 0.1%, M: 0.5%
Annotation			
Proportion repeat (%)	7.93	7.85	21.95
Proportion coding,^c^*n* (%)	22.6 (37.09)	19.1 (32.18)	28.8 (28.68)
Proportion intron, *n* (%)	16.6 (27.29)	16.6 (27.9)	33.1 (33.02)
Number of protein- coding genes	16,367^d^	14,626	20,184
Proteome BUSCO^b^	C: 90.2% [S: 87.5%, D: 2.7%], F: 1.9%, M: 7.9%	C: 89.1% [S: 86.2%, D: 2.9%], F: 2.0%, M: 8.9%	C: 100% [S: 99.6%, D: 0.4%], F: 0%, M: 0%

MT, mitochondrial genome.

a See https://parasite.wormbase.org/Caenorhabditis_elegans_prjna13758/Info/Index/.

b BUSCO scores assessed against nematoda_odb10 (*n* = 3131); reported as C = complete, S = single copy, D = duplicated, F = fragmented, and M = missing.

c Using the longest isoform per gene.

d Including 698 loci with similarity to mobile elements.

### Comparing chromosome structure in *O. tipulae* and other nematodes

**Figure 2:**
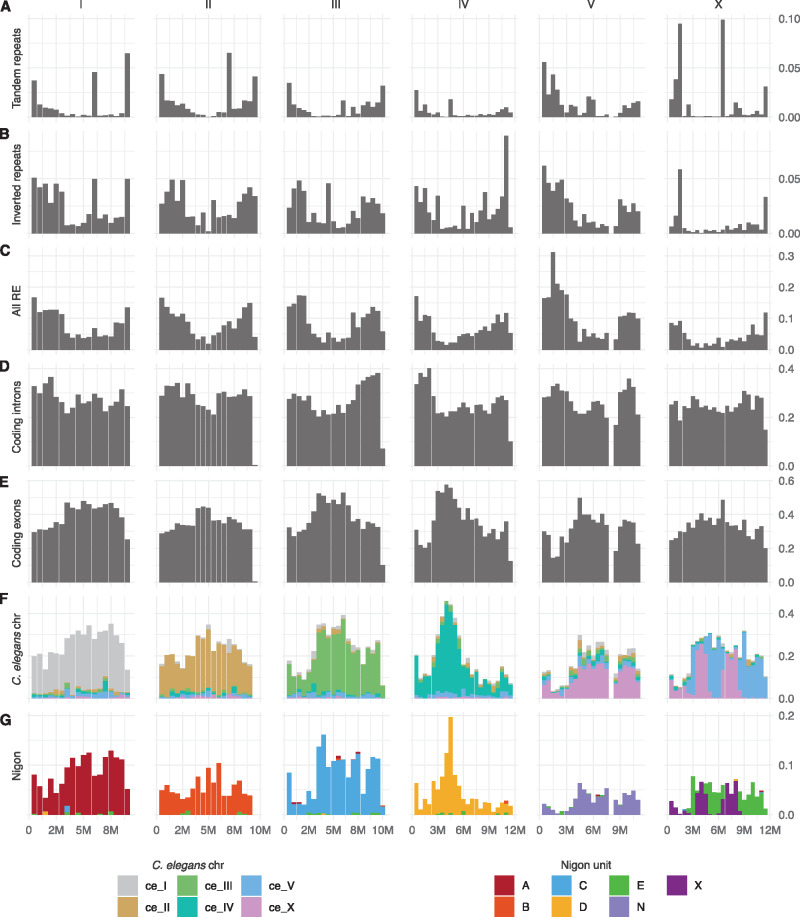
The *O. tipulae* CEW^1^ genome. Feature densities along *O. tipulae* chromosomes per 500-kb non-overlapping windows. (A) Tandem repeats identified by TRF. (B) Inverted repeats identified by IRF. (C) All repetitive elements annotated as described in the Materials and Methods section. (D) Intron of protein-coding genes. (E) Exons of protein-coding genes. (F) Exons of genes with reciprocal best hits to *C. elegans* genes with bins filled according to the gene location in *C. elegans*. (G) Exons of genes classified as Nigon loci with bins filled according to their Nigon element classification.

A striking feature of the *C. elegans* genome is the strong patterning of genic and non-genic features along each chromosome (C. elegans Sequencing Consortium 1998). Repeats are more abundant on the autosome arms and are largely excluded from the chromosome centers, while GC proportion has higher variance in the arms. The *C. elegans* X chromosome has a similar pattern albeit less pronounced. These patterns are likely to be generated by the local recombination rate, which is high on autosome arms and lower in the autosome centers and on the X chromosome (Rockman and Kruglyak 2009). We explored the chromosomal *O. tipulae* genome for similar patterns. The arms of *O. tipulae* chromosomes show a higher repeat and intronic sequence fraction and a lower exonic fraction compared to the centers (Figure 2). The pattern of GC proportion along *O. tipulae* chromosomes also matched expectations from *C. elegans* (and other species, Supplementary Figure S6), except for two regions. Both Otip_IV and Otip_V have regions where there is a step change in GC proportion (Figure 1A, inner circle). The boundaries of these step changes were examined and were supported by a normal (320 fold) coverage of PromethION reads. We interpret these as recent inversions where background processes have not yet restored the local GC proportion pattern.

**Figure 3:**
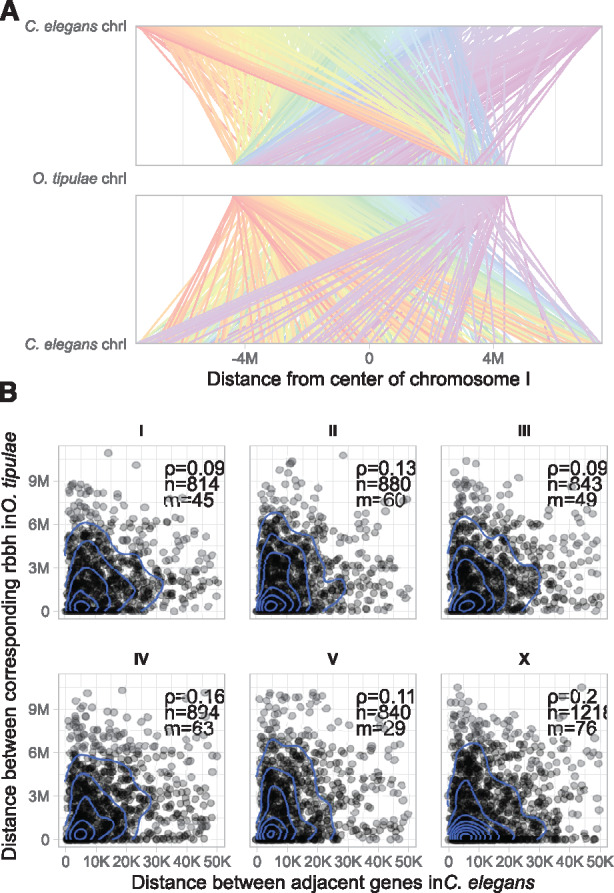
Local gene order comparison between *O. tipulae* and *C. elegans*. (A) Whole chromosome comparison of *O. tipulae* chromosome I (Otip_I) and its homolog, *C. elegans* chromosome I (Cele_I). Lines link loci that are reciprocal best-BLAST hits. In the top panel, the lines are colored by their placement on *C. elegans* Cele_I (a spectrum from red on the left arm through to purple on the right arm). In the lower panel, the lines are colored by their placement on *O. tipulae* Otip_I (a spectrum from red on the left arm through to purple on the right arm). (B) Gene neighborhoods are not conserved between *O. tipulae* and *C, elegans*. We measured the separation distance between each pair of neighboring *C. elegans* loci for which we could identify single-copy reciprocal best-BLAST relationships between *O. tipulae* and *C. elegans* and plotted the separation between these ortholog pairs in *C. elegans* (*x*-axis) and *O. tipulae* (*y*-axis) for each chromosome. Genes associated to an operon in *C. elegans* were discarded from this analysis. Correlation tests had *P*-values <0.01 for all except chromosomes I and III. Ortholog pairs more than 50 kb apart in *C. elegans* were excluded. 2D kernel density (blue lines) was visualized using ggplot2::geom_density_2d. ρ: Spearman's rank correlation coefficient; *n*: number of orthologous pairs including pairs excluded from the plot; *m*: the number of pairs excluded from each panel.

As expected from comparisons within the genus *Caenorhabditis* (Slos *et al*. 2017; Stevens *et al.* 2020) and between *Caenorhabditis* and the strongylomorph *H. contortus* (*Doyle et al. 2020*), while neighboring orthologous genes tended to be found on the same linkage groups in *O*. *tipulae* and in *C. elegans*, local gene neighborhoods were not highly conserved (Figure 3). Interestingly, genes in the centers of the *C. elegans* autosomes were not more likely to be retained in the centers of *O. tipulae* chromosomes, and gene neighborhood conservation was largely absent apart from conserved operonic gene sets.

### Chromosomal elements are conserved across the Rhabditida

**Figure 4:**
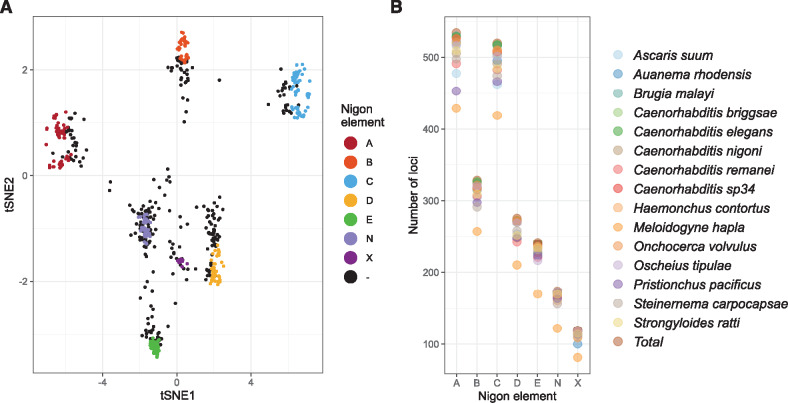
Loci that define Nigon elements in rhabditid nematodes. (A) t-SNE plot of the Gower distance between 3412 orthologous gene families mapped to the chromosomal assemblies of nine species. The 2191 loci that are included in the Nigon element sets are colored. The black dots represent loci not assigned to a Nigon unit. Parameters used for t-SNE are perplexity = 500, max_iter = 1000, initial_dims = 50, and theta = 0.5. (B) For each Nigon-defining set of loci, we counted the number of loci found in each species. The total number of loci per set is indicated by a star. The assembly of *M. hapla* has the fewest loci and lowest proportion of loci in all Nigon sets.

Previously, we defined conserved nematode chromosomal elements, called Nigon elements, through manual comparison of five genomes from species in Rhabditina (Clade V) (Tandonnet *et al.* 2019). Manual generation of chromosome assignments is not sustainable, and piecewise addition will fossilize initial taxonomic and data biases. We therefore developed an objective, algorithmic method to identify and group loci that define conserved elements based on shared chromosomal colocation (Figure 4A). Using this method, we were able to include all available rhabditid nematode genomes that have been reported to be chromosomally complete or near complete. We identified 2191 loci that had a one-to-one orthologous relationship between most species. These formed seven clusters of loci colocated on chromosomes in most species. These clusters of loci were used to paint the nematode chromosomal assemblies and replicate our previous, manual definition of Nigon elements. Then, clusters had between 534 loci (defining Nigon element A) and 119 (defining NigonX) (Figure 4B and Supplementary Table S10). The *M. hapla* genome had low BUSCO scores and low representation of orthologs in all the Nigon element sets.

### Chromosome evolution and homology in the Rhabditida

**Figure 5:**
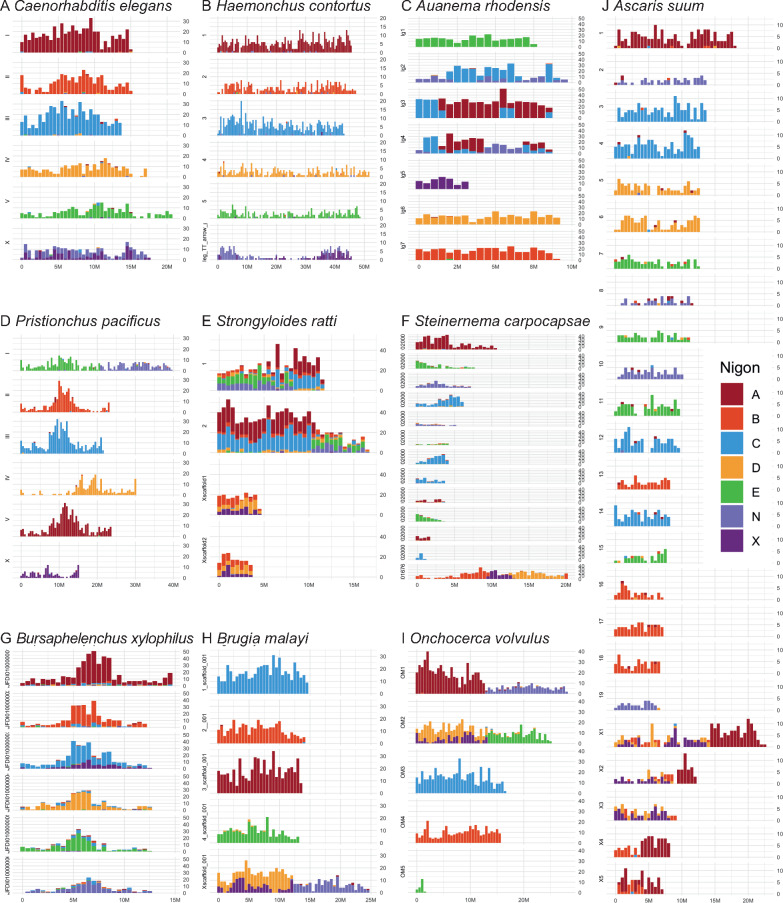
Nigon painting of rhabditid nematode chromosomes. Examples of nematode chromosomal assemblies painted by their content of Nigon-defining loci. Each subgraph shows the count of loci mapped in non-overlapping 0.5 Mb windows along a chromosome as a stacked histogram colored by Nigon origin. The *X*- and *Y*-axes are scaled to the maxima within a species in each panel (X: chromosome length, Y: Nigon-defining loci per interval) within each species. The legend in panel J applies to all nine chromosome panels. (A) *C. elegans* (Rhabditina, Rhabditomorpha), (B) *Haemonchus contortus* (Rhabditina, Rhabditomorpha), (C) *A. rhodensis* (Rhabditina, Rhabditomorpha), (D) *P. pacificus* (Rhabditina, Diplogasteromorpha), (E) *S. ratti* (Tylenchina, Panagrolaimomorpha), (F) *S. carpocapsae* (not a fully chromosomal assembly; Tylenchina, Panagrolaimomorpha), (G) *B. xylophilus*, (H) *B. malayi* (Spirurina, Spiruromorpha), (I) *O. volvulus* (Spirurina, Spiruromorpha), (J) *A. suum* (Spirurina, Ascaridomorpha). Panel (K) shows the color key for the other panels.

**Figure 6:**
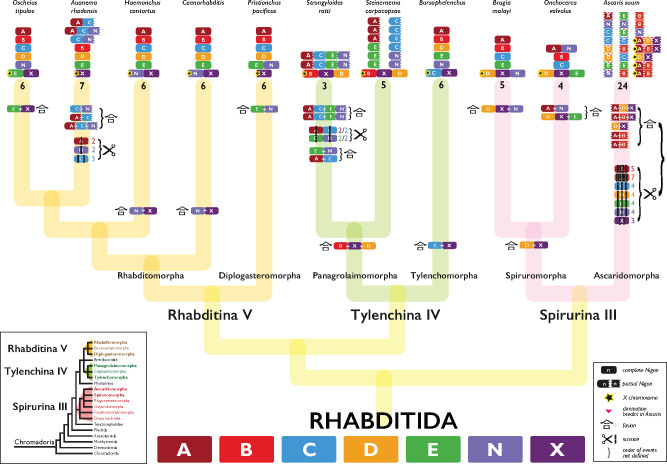
A model of chromosome evolution in Rhabditida. For each species, the classification of chromosomes to Nigon elements is shown (see Figures 2 and 5 and Supplementary Figure S7), including rearranged chromosomes. X chromosomes are indicated by a star. For each lineage, we have inferred the patterns of chromosome scission (the 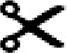 symbol; the number indicates the number of fragments resulting) and fusion (the Chinese/Kanji symbol for “fusion point” 
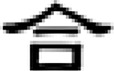
) on the tree. Where the order in time of events is not resolved, we have bracketed them. In *A. suum*, a pink triangle indicates positions of internal cleavage of germline chromosomes during diminution. Note that the “partial Nigons” in *S. carpocapsae* are assembly scaffolds rather than chromosomes. The cladogram representing the phylogeny of Chromadoria nematodes (inset, lower left; Blaxter and Koutsovoulos 2015) is derived from the phylogenetic analysis of shared protein-coding genes.

The general conservation of chromosome number (*n* = 6) in rhabditid nematodes might suggest that these karyotypes reflect a static pattern of locus colocation and Nigon element structure. Previous analyses suggested that this was not the case (Rödelsperger *et al*. 2017; Tandonnet *et al.* 2019), and we have here used chromosome painting to define Nigon element structure in each species (Figures 2 and 5 and Supplementary Figure S7) and to build a model of rhabditid karyotype evolution (Figure 6). We recapitulated previous findings made on a limited set of species (Tandonnet *et al.* 2019) and extended the Nigon element schema to species across Rhabditida. In general, Nigon elements were found as independent chromosomes across Rhabditida, but many species showed patterns of Nigon element-defining locus mixing that evidenced past fusions and breakages.

In Rhabditina, all seven of the Nigon elements were present as distinct chromosomes in at least one species. A relatively limited number of fusions and scissions were necessary to explain the observed patterns of Nigon assignment. For example, in *P. pacificus* Ppa-chrI was identified as a recent fusion of NigonA and NigonN (Figure 5D). As noted previously (Rödelsperger *et al.* 2017; Tandonnet *et al.* 2019), Ppa-chrI retains structural signal of two chromosomal elements, with a dual pattern of repeat density and gene density peaks, and these features are consistent with the NigonA and NigonN portions of this chromosome. The *P. pacificus* X chromosome contained only loci from NigonX. Nigon painting of the *Caenorhabditis* species and *H. contortus* showed that the *n* = 6 karyotype of these species is derived from the seven Nigon elements through fusion of NigonN with NigonX to form the X chromosome (Table 3). In contrast to the NigonA–NigonN fusion in *P. pacificus*, the NigonN and NigonX loci in the X chromosomes of *Caenorhabditis* were intermixed, suggesting that processes of intrachromosomal rearrangement have removed evidence of distinct NigonN or NigonX domains.

**Table 3:** Nigon elements and the X chromosomes of rhabditid nematodes

	Nigon is distinct in at least one species in			Nigon is part of X chromosome in											
	Rhabditina	Tylenchina	Spirurina	Rhabditina					Tylenchina				Spirurina		
				*Ot*	*Ar*	*Ce*	*Hc*	*Pp*	*Sr*	*Sc*	*Bx*	*Bo*	*Bm*	*Ov*	*As*
NigonA	Yes	Yes	Yes												Yes
NigonB	Yes	Yes	Yes						Yes	Yes					Yes
NigonC	Yes	Yes	Yes								Yes	Yes			
NigonD	Yes	Yes							Yes	Yes			Yes	Yes	Yes
NigonE	Yes	Yes	Yes	Yes										Yes	
NigonN	Yes	Yes	Yes			Yes	Yes						Yes		
NigonX	Yes			Yes	Yes	Yes	Yes	Yes	Yes	Yes	Yes	Yes	Yes	Yes	Yes

This NigonX–NigonN fusion was not observed in other Rhabditina species. In *O. tipulae*, NigonX was found to have fused with NigonE to form the X chromosome, and the blocky pattern of locus distribution suggested that the fusion was more recent than the NigonN–NigonX fusion in *Caenorhabditis* and *H. contortus* (Figure 5, A and B). In *A. rhodensis*, the loci defining NigonX were all found on Arh-lg5, which is the X chromosome (Figure 5C). NigonA, NigonC, and NigonN loci were distributed, with a blocky pattern, across three *A. rhodensis* autosomes (Arh-chr2, Arh-chr3, Arh-chr4), suggesting a relatively recent set of scission and fusion events (Figure 5C). *H. contortus* and the genus *Caenorhabditis* have similar Nigon element classification of their chromosomes, and in particular both have an X chromosome that is a fusion of NigonN and NigonX. While this might suggest a shared, ancestral NigonN–NigonX fusion, molecular phylogenies robustly place *H. contortus* closer to *O. tipulae* and *A. rhodensis* than to *Caenorhabditis*, implying that the NigonN–NigonX fusions in these two groups were independent. An analysis of the non-chromosomal genome assemblies of three additional species, *H. bacteriophora* (an insect pathogen more closely related to *H. contortus*), *D. coronatus* (Hiraki *et al.* 2017), and *D. pachys* (Fradin *et al.* 2017) (free-living species closely related to *Caenorhabditis*), assists in resolving this issue. *D. coronatus* has a karyotype of *n* = 1, but the assembly (scaffold N50 of 1 Mb) shows this chromosome retains residual signal of its origin through fusion of separate ancestral chromosomes in the form of regional similarity to *Caenorhabditis* chromosomes. This is also evident in the lower-contiguity *D. pachys* assembly (Fradin *et al.* 2017). Nigon painting of the longest scaffolds of *D. coronatus* identified several with majority NigonX content and no NigonN loci, and several with majority NigonN content and no NigonX loci (Supplementary Figure S7). Only one long scaffold had both NigonN and NigonX loci. This suggests that NigonN and NigonX were distinct entities before they fused with the rest of the genome in *Diploscapter* and by inference were distinct in the last common ancestor of *Diploscapter* and *Caenorhabditis*. In the *H. bacteriophora* assembly (scaffold N50 of 312 kb; Bai *et al.* 2013), there were no scaffolds that carried loci from both NigonN and NigonX, and several carried solely NigonN or NigonX loci (Supplementary Figure S7). While the *H. bacteriophora* genome assembly is not chromosomal, this pattern indicates distinct domains of NigonN and NigonX ancestry and argues against the presence of an ancestral fusion in the last common ancestor of *H. bacteriophora* and *H. contortus*. We note that on the *H. contortus* X chromosome the NigonN loci are clustered toward one end and the NigonX loci toward the other (Figure 5B), which we interpret as a relict pattern arising from the NigonN–NigonX fusion. This partitioning is not observed in *Caenorhabditis* species (Figure 5A andSupplementary Figure S7). Thus, at the base of Rhabditina, we predict that there were seven distinct linkage groups, corresponding to Nigon elements A through E, N, and X (Figure 6).

In Spirurina, NigonA was an independent autosome in *B. malayi* (*Bma-chr3*) and a NigonA element was identified as recently fused with a NigonN element in *O. volvulus* to form *Ovo-OM1* (Figure 5, H and I, and Supplementary Figure S7). Chromosomes consisting nearly completely of NigonA loci were found in *A. suum* (*Asu-chr1*) (Figure 5J). NigonB is an independent autosome in both *B. malayi* and *O. volvulus*, and four *A. suum* autosomes are painted only by NigonB loci (Figure 5, H–J). In *A. suum*, some NigonB loci are also found on the X chromosomes. Similarly, NigonC loci paint independent chromosomes in *B. malayi* and *O. volvulus* and four autosomes in *A. suum.* NigonN appeared to have fused recently and independently in *B. malayi* (with NigonD to form the X chromosome) and *O. volvulus* (with NigonA to form *Ovo-OM1*). That these fusions are recent is supported by patterns of repeat and GC content across the chromosomes. In *A. suum*, NigonN loci are largely restricted to four NigonN autosomes. The X chromosomes of *A. suum*, *B. malayi*, and *O. volvulus* each carried evidence of a fusion between NigonD and NigonX. It was not possible to discern whether this fusion was ancestral to Spirurina or arose independently in the Ascarididomorpha and Spiruromorpha (Figure 6). Independent fusion was supported by the finding that there are distinct *A. suum* autosomes only painted by NigonD, that NigonX loci while wholly limited to the five *A. suum* X chromosomes are not always associated with NigonD loci, and that NigonD and NigonX loci form distinct blocks in the *A. suum* X chromosomes. On *A. suum* Asu_chrX1, the NigonD and NigonX domains are resolved as distinct somatic chromosomes by chromatin diminution. In *B. malayi* and *O. volvulus*, the NigonD and NigonX loci were intermixed, suggesting long-term association and mixing by intrachromosomal rearrangement.

It is notable that while all the autosomes of *A. suum* were painted by loci from a single Nigon element set, all five X chromosomes had mixed origins, involving blocks of NigonA, NigonB, NigonD and NigonX loci. The chromosomes of *A. suum* are subject to chromatin diminution in somatic cells of the embryo (Wang *et al.* 2020). This process generates remodeled telomeres for all chromosomes, and specific cleavage at internal sites in some chromosomes such that somatic cells have more chromosomes (but less genetic material overall) than do germline cells. It is striking that all but one of the intrachromosomal cleavage points were between blocks of chromosome with distinct Nigon identity. The one cleavage not between Nigon blocks (in *Asu-chr1*) separated two NigonA components. Not all blocks of Nigon identity were separated by cleavage during diminution (Figure 6).

In Tylenchina, retention of ancestral units, breakages, and fusions also describe the present day chromosome structures observed (Figure 5, E–G). Both *Bursaphelenchus* species have six chromosomes and displayed similar Nigon element patterning. Five chromosomes were resolved as containing loci from a single Nigon element each, and one chromosome, the *Bursaphelencus* X chromosome, was an intermixed fusion of NigonC and NigonX. In *S. carpocapsae*, which is not fully chromosomally assembled, the 12 autosomal scaffolds, which correspond to four autosomes, each had a single Nigon identity. We predict that these scaffolds will assemble to yield four autosomes corresponding to NigonA, NigonC, NigonE, and NigonN. The *S. carpocapsae* X chromosome comprised three domains corresponding to NigonB, NigonX, and NigonD (Figure 5F). *S. ratti* has two autosomes and an X chromosome. The autosomes were modeled as being a complex product of a two stage fusions-scissions-fusions process. The first fusions were between NigonA and NigonC and between NigonE and NigonN. After some time (as evidenced by the intermixing of loci), these fusion chromosomes were split and refused to form Sr-chr1 and Sr-chr2. The second set of scissions and fusions was relatively recent, as each autosome had distinct domains corresponding to the presumed ancestral fusions. The *S. ratti* X chromosome was painted by loci corresponding to NigonB, NigonD, and NigonX (Figure 5E), and the NigonB, NigonD, and NigonX loci were fully intermixed. While NigonX was not found as a distinct chromosome in any of the four tylenchine species, the distinct fusions of NigonX in *Bursaphelenchus* (with NigonC) and the other species (with NigonD and NigonB) indicated that this element was a distinct entity at the base of Tylenchina. It is notable that NigonD and NigonX are independently associated with the X in some Tylenchina and in Spirurina. *M. hapla* has 17 chromosomes, but Nigon painting of these did not yield definitive Nigon assignments (Supplementary Text 2). We noted that there was no association between NigonD and NigonX loci on the *M. hapla* chromosomes.

The independent existence in a common ancestor of Rhabditina, Tylenchina, and Spirurina of elements corresponding to Nigons A, B, C, D, E, and N was evident from the identification of chromosomes, or distinct chromosome domains, in all three suborders corresponding to these elements. The NigonX element was always found on the X chromosome, frequently paired with other elements. NigonD and NigonX were not associated in Rhabditina but were variably associated in Tylenchina and Spirurina, likely due to convergent fusion events (Figure 6).

### Dynamic evolution of rhabditid sex chromosomes

NigonX was the only element consistently associated with the sex chromosome all the nematode species analyzed (Table 3). In addition, the NigonX element was much more likely to be involved in fusions with other Nigon elements. The histories of loci defining NigonD and NigonX were intertwined in both Tylenchina and Spirurina. In *S. ratti* and *S. carpocapsae*, the X chromosomes were ancestral fusions of NigonB, NigonD, and NigonX, and this fusion was fully intermixed in *S. ratti* (Figure 5E) but unmixed in *S. carpocapsae* (Figure 5F), while in *Bursaphelenchus* species, the NigonX element was intermixed with NigonC. NigonX loci were found on three of the five *A. suum* X chromosomes (As-chrX1, As-chrX2, and As-chrX3), mixed or fused with other Nigon element loci (Figure 5J). The other two *A. suum* X chromosomes (As-chrX4 and As-chrX5) are fusions of NigonA and NigonB loci. The NigonA loci tended to form distinct domains in all the *A. suum* X chromosomes, which suggest relatively recent fusion, but the NigonD and NigonX loci were intermixed. *A. suum* also had two autosomes that contained only NigonD loci (As-chr5 and As-chr6). In both spiruromorph nematodes, a NigonD plus NigonX intermixed domain was found in the X chromosome, but this had fused with different, autonomous Nigon elements in *B. malayi* (with NigonN) and *O. volvulus* (with NigonE) (Figure 5, H, I). The complete admixture of NigonD and NigonX loci in the spiruromorph X chromosome domains suggests ancient fusion, especially since the *B. malayi* and *O. volvulus* genomes are largely collinear both within non-fused chromosomes and within fused chromosome blocks (Foster *et al.* 2020). Because NigonX and NigonD are present, intermixed, in the X chromosomes of both Ascaridomorpha and Spiruromorpha, the NigonD–NigonX fusion could be ancestral to Spirurina. However, the presence of NigonD-only autosomes in *A. suum* suggested that NigonD was present as an independent element in this lineage, and we model these NigonD–NigonX fusions as independent events (Figure 6).

NigonD was also present in the X chromosomes of some Tylenchina, as part of a NigonB–NigonD–NigonX fusion. In *S. carpocapsae*, this fusion appeared to be recent, as the three sets of Nigon element-derived loci occupied distinct domains, with NigonX central (Figure 5F). In *S. ratti*, the loci from NigonB, NigonD, and NigonX were intermixed on *Sra-chrX*, but some NigonD loci were found on the two autosomes (Figure 5E). These autosomal NigonD loci were found in association with some NigonB loci, perhaps as a result of translocation from an ancestral intermixed NigonB–NigonD chromosome. NigonD was an independent autosome in the *Bursaphelenchu*s species, and along with the distinctness of the NigonD domain within *S. carpocapsae* Sc_X, this suggests that NigonD was an independent element in Tylenchina also. The NigonB–NigonD–NigonX fusion is, we suggest, an association of NigonD and NigonX independent of that in Spirurina.

### Functional analysis of Nigon element loci

The distinct histories of the gene sets associated with each Nigon element means that these sets of loci have been linked for a significant period of time and may have been selected to stay together or evolved to collaborate. We explored whether such association might reflect the shared biological function of these genes. We interrogated functional enrichment of the Nigon-defining loci through the KEGG pathway annotation of *C. elegans* orthologs. We first compared KEGG pathway annotations of Nigon-defining loci to those of the full gene set of *C. elegans* (Supplementary Table S11). Terms relevant to RNA transport were enriched in NigonA loci and NigonC loci, ribosome biogenesis was enriched in NigonA loci and spliceosome pathway was enriched in NigonC loci. ErbB signaling and calcium signaling pathways were enriched in the NigonD locus set. In NigonN loci, Hippo signaling was enriched. In NigonX loci, annotations relevant to axon regeneration, calcium signaling pathway and neuroactive ligand–receptor interaction were significantly enriched. No KEGG pathways were enriched among loci of NigonB or NigonE. The Nigon-defining loci were drawn from a specific, conserved subset of all *C. elegans* loci, and this conservation will have *a priori* biased the annotations being assessed. We also compared the annotations associated with each Nigon loci set against all 2175 Nigon loci and only detected enrichment in NigonX loci, in the KEGG axon regeneration pathway (enrichment significance 4.40e10 − 4).

## Discussion

### New technologies and complete genome sequencing of *O. tipulae*

New sequencing technologies and the development of improved assembly toolkits are generating more highly contiguous reference genome assemblies. Here, we use the Oxford Nanopore long reads to generate chromosomally complete contigs representing all the nuclear chromosomes and the mitochondrion of the free-living nematode *O. tipulae*. While the data are sufficient to generate a chromosomally contiguous assembly, not all assembly tools were able to generate this from the data, and there is evidently still development work to be done. Alternate methods of generating single molecule, long read data such as the Pacific Biosciences SEQUEL II CLR (single pass) and HiFi (circular consensus, multiple pass), have similar properties to PromethION data, with the HiFi standing out as having higher per-base accuracy. This higher per-base accuracy likely simplifies the assembly process, in particular in the resolution of repeat structures that are close to but not 100% identical. However, the PromethION data, which can include very long reads, may be better at traversing recent segmental duplications and homogenized multicopy loci (Nurk *et al*. 2020). In our assembly of *O. tipulae*, the only identified remaining collapsed repeat was the 6.8-kb repeat of the ribosomal RNA cistron (nSSU, 5.8S, and nLSU loci), which we estimate is repeated about 117 times, summing to 801 kb. This repeat is homogenized, and thus, only very long-range technologies, such as ultralong nanopore reads or BioNano mapping, could resolve it fully.

One unexpected and striking feature of the *O. tipulae* genome is the structure of the telomeres. The PromethION data robustly predict two telomere repeat addition sites at each end of each chromosome, generating a core, high coverage chromosome with lower coverage subtelomeric extensions at each end. The extensions are supported by unique mapping of independently generated short Illumina data. Nearly 350 kb (or 0.6% of the genome) is in these extensions, which carry additional, expressed protein-coding genes. We currently interpret the extensions as segments of chromosomes that have been specifically removed from the genomes of a proportion of cells in each nematode, possibly through developmentally regulated chromatin diminution. The presence of helitron mobile elements specifically in the subtelomeric elements is intriguing. The helitrons contain nuclease and Pif1 DEAH-box helicase domains. In yeast and other taxa, Pif1 helicases are intimately involved in DNA metabolism, and in particular, in DNA replication and telomerase function and regulation. It is possible that the *O. tipulae* telomeric helitrons are parasitic elements that have generated extended subtelomeric regions in which they reside and that they also control the excision of these telomeric extensions and the specific addition of neo-telomeres. Alternatively, the nematode may have co-opted the helitrons to regulate a chromatin diminution process that regulates expression of germline-restricted genes by eliminating them from the soma, as in *A. suum* (Wang *et al*. 2012). In nematodes, chromatin diminution distinguishing soma from germline has been described in Ascaridomorpha and in XX and X0 sperm made by parthenogenetic female *S. papillosus* (Wang and Davis 2014). Given that the diminution signal we observe is present on all chromosome ends, we currently favor an ascaridomorph-like process that distinguishes a germ-line genome from a somatic one. It must be distinct from the ascaridomorph process, as the internal breakage and addition sites in *A. suum* are associated with multi-kilobase regions while the *O. tipulae* sites are precise. It may be that similar processes are present in other nematodes, and other species, but have been overlooked because of the lack of contiguity of the previously available short-read sequence data.

### Evolution of rhabditid nematode karyotypes

We have refined an approach to defining loci that define conserved linkage groups. In many taxa, it is possible to use gene neighborhoods (gene order and synteny) to drive inference of ancestral karyotypic organization (Kim *et al.* 2017). However, we and others have noted that gene order is poorly conserved in rhabditid nematodes (Stein *et al.* 2003; Teterina *et al.* 2020). This is interesting because deeply evolutionary conserved genes in of *C. elegans* tend to be found in chromosomal centers and novel loci in the arms (C. elegans Sequencing Consortium 1998). Despite this, we observed other chromosomal features that were similar to *C. elegans*, such as the differential abundance of repeats on the presumed arms of *O. tipulae* autosomes. This suggests that distinct evolutionary processes may drive these patterns.

We were able to derive sets of loci that traveled together on linkage groups through rhabditid genome evolution by clustering orthologs based on a numerical representation of their chromosomal location in each species. This process is robust, and extendable to incorporate additional genomes. It is also applicable to other taxa where chromosomally complete genomes are available. In Rhabditida, we identified seven clusters of loci that define seven chromosomal units, named Nigon elements. These elements are fully congruent with a previous manual estimate (Tandonnet *et al.* 2019). Painting the chromosomal genome assemblies of 14 rhabditid species revealed that in no species were all of these elements present as distinct chromosomes, but each Nigon element was found as a distinct element in several species. The NigonX element was more likely to be involved in fusions than the other elements, and these fusions were identified as the X sex chromosome (Table 3). In species in Rhabditina, the NigonX element was fused with NigonE and NigonN, in Tylenchina with NigonB, NigonC and NigonD, and in Spirurina with Nigon D, NigonE, and NigonN. We were not able to apply the Nigon element model to *M. hapla*, where mapping to the 17 chromosomal scaffolds yielded only a few with majority assignment to one element. It will be informative to explore chromosomal evolution in the plant parasitic Heteroderidae further.

**Figure 7:**
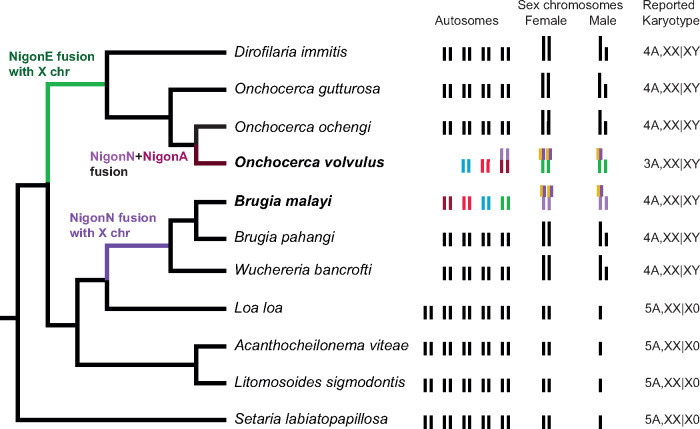
Sex chromosome evolution in the filarial nematodes (Spiruromorpha). Within the filarial nematodes, karyotypes have been determined for a number of species. The phylogeny (cladogram to the left) is derived from multilocus phylogenomic analysis and is in agreement with marker gene-based phylogeny (Lefoulon *et al.* 2015). The karyogram and karyotype data are from the work of Post (2005). The inferred position of fusion events within the filarial phylogeny is indicated, and the karyotypes of *O. volvulus* and *B. malayi* are colored by their Nigon assignment (see Figures 5, H and I, and 6).

*Brugia* and *Onchocerca* are unusual in Spiruromorpha in having an apparent XX:XY sex determination system (Post 2005). Within the filarial nematodes, an XY system has evolved twice from an ancestral XX:X0 system, once in the ancestor of *Onchocerca* and *Dirofilaria* species and once in the ancestor of *Wuchereria* and *Brugia* species (Figure 7) (Post 2005). It was proposed from karyotypic analyses that the neo-X chromosome in *Onchocerca* and *Brugia* arose from the fusion of an autosome with the ancestral X and that the neo-Y chromosome in these species was just this autosomal chromosomal component (Post 2005). Our analysis of Nigon element conservation supports this model but additionally suggests that the enlarged X chromosomes in the two species are the results of two distinct fusions with an ancestral X: in *B. malayi* with NigonN and in *O. volvulus* with NigonE. Foster *et al.* (2020) have argued, based on the presence on spiruromorph X chromosomes of NigonD loci (*i.e.* loci mapping to *C. elegans* chromosome IV) that NigonD was the ancestral sex determination element of all Rhabditida and that sex determination function transitioned to NigonX only later in rhabditid evolution. Foster *et al.* were unable to identify NigonN. We think it more parsimonious to retain a model where NigonX is the sex-determining element, and other Nigon elements variably associate with it. NigonD is not part of the X chromosome in *Bursaphelenchus* or in any rhabditine nematode, and some autosomes in *A. suum* contain only NigonD loci.

While males in *B. malayi* and *O. volvulus* have a pair of sexually dimorphic chromosomes it is not clear whether the “Y” chromosome actually carries a male-determining locus, or whether the system is a modified Xo system, as is found in the tylenchine *S. papillosus* (Albertson *et al.* 1979). In *S. ratti*, sex determination is XX:Xo, and the X chromosome is an intermixed fusion of NigonB, NigonD, and NigonX. In the related *S. papillosus*, sex determination is also XX:Xo, but in this species haploidy of X is generated by intrachromosomal chromatin diminution. While the available genome assembly for *S. papillosus* is not chromosomal, mapping of genetic markers indicated that the part that is lost is likely to be the NigonB–NigonD–NigonX component, while the remainder of the *S. papillosus* X appears to be homologous to *S. ratti* chromosome I (Nemetschke *et al*. 2010a). Thus, *S. papillosus* male-determining sperm have a “reduced-X” chromosome that contains only the autosomal part, and males are still diploid for the autosomal part. So are the *B. malayi* and *O. volvulus* sex determination systems XX:XY or apparent XY systems that biologically behave as XX:Xo? Read data from males and females identified only a very small segment of genome (2.7 Mb in many short contigs) that was unique to male *B. malayi*, and a previously identified, male-linked locus (transposon on Y, TOY) was part of this male-limited genome. Karyotypic analyses identified the *B. malayi* X chromosome as being similar in size to the X chromosomes of XX:X0 species, and similar in size to the autosomes (Post 2005). We interpret this sequence (and TOY) as being repeat accumulation in the subtelomeric regions of the short-form X chromosome that was lost consequent to the fusion event between the ancestral X and NigonN chromosomes and doubt that it has roles in sex determination. The NigonN component of the *B. malayi* X is diploid in males and females while the 12-Mb NigonD–NigonX portion is haploid in males.

Other spiruromorphs, including close relatives of *Onchocerca* and *Brugia*, have an XX:X0 sex determination system (Post 2005), suggesting that this mode is ancestral. This pattern leads us to question whether sex determination in *B. malayi* and *O. volvulus* is in fact XX:XY, where, by analogy to XX:XY systems in other taxa, a sex (male) determining locus is present on the Y. In *B. malayi* and *O. volvulus*, where the non-NigonD–NigonX component of the X chromosome derives from fusion with different chromosomes, we propose that the same haploid-X mechanism operates and that the apparent XX:XY sex determination system is in fact an XX:X0 system, where the additional component (NigonN in *B. malayi* and NigonE in *O. volvulus*) is always diploid. This must mean that the fusion partner, diploid in males and females, must be under distinct dosage compensation control compared to its sex-determining partner, as is likely the case in *S. papillosus*. Similar sex chromosome fusions where the newly fused parts have distinct dosage compensation mechanisms have been identified in another species with holocentric chromosomes, the butterfly *Danaus plexippus*. In *D. plexippus*, the neo-Z chromosome has two distinct modes of compensation, spatially distributed along the fusion based on the origin of the segment (*i.e.* expression of the Z component is halved in ZZ males, while expression of the autosomally derived fragment is doubled in WZ females; Gu *et al.* 2019).

### Functional coherence of loci that define Nigon elements

As these Nigon-defining loci have been colocated on the same karyotypic unit for much of rhabditid nematode evolution, we wondered whether each set had a functional coherence such that the colocated genes functioned together in specific pathways. We identified functional enrichment in five of the seven gene sets in *C. elegans* when the whole *C. elegans* gene set was used as a comparator. As we selected these genes based on their largely one-to-one conservation across Rhabditida, it would be expected that they would be enriched in conserved function, and so this result is perhaps not surprising. When using only the set of Nigon-defining loci as a reference, however, we found functional enrichment only in NigonX-linked loci. Based on our model, the loci that define NigonX have been on the sex determination chromosome of rhabditid nematodes since the last common ancestor of Tylenchina, Spirurina, and Rhabditina. These loci will thus have been exposed as haploid in males and will have had an effective population size of ∼0.75 of the size of any autosomal locus for the entirety of rhabditid evolution. Genes with essential functions are more rarely found on the X chromosome than in the autosomes of *C. elegans*, while genes with non-lethal, post-embryonic knock-down phenotypes are enriched in the X chromosome (Kamath *et al.* 2003).

### Outlook

The telomere-to-telomere chromosomal assembly of *O. tipulae* can now stand as a platform for future work on the developmental and population genetics of this important model species. Investigation of the biological importance of the telomeric extensions and especially of their presence or absence in other species is of particular importance. Why do some genes stay on the same chromosome, while others appear to move freely? What mechanisms drive the processes of chromosomal structure, and why do some species have distinct patterns of intra- and inter-chromosome rearrangement? In Lepidoptera, there is a general conservation of karyotype (with *n* = 31) and genes tend to be situated on homologous chromosomes in the same order (*i.e.* there is strong conservation of micro- and macro-synteny), but some taxa diverge strongly from this pattern and have very different chromosome numbers (from *n* = 4 to >200) (de Vos *et al.* 2020) that may not be simply described by fusion of whole chromosomes, or scission of chromosomes into multiple parts (Hill *et al.* 2019). In the Lepidoptera, karyotypic change is associated with speciation (de Vos *et al.* 2020), but whether this is also true in nematoda is not clear, though we note the relatively rapid karyotypic evolution in filarial nematodes (Figure 7) and the existence of genera and orders such as *Diploscapter* in Rhabditina (*n* = 1–7) (Fradin *et al.* 2017) and the Ascaridomorpha (*n* = 1–24), where chromosome counts vary greatly (Walton 1959). We look forward to an increase in chromosomal assemblies from rhabditid and other nematodes in the near future to further explore patterns and processes in nematode chromosome evolution.
